# The Neurobiology of Imagination: Possible Role of Interaction-Dominant Dynamics and Default Mode Network

**DOI:** 10.3389/fpsyg.2013.00296

**Published:** 2013-05-24

**Authors:** Luigi F. Agnati, Diego Guidolin, L. Battistin, G. Pagnoni, K. Fuxe

**Affiliations:** ^1^IRCCS San Camillo, Venice, Italy; ^2^Department of Molecular Medicine, University of Padova, Padova, Italy; ^3^Department of Biomedical Sciences and Neuroscience, University of Modena, Modena, Italy; ^4^Department of Neuroscience, Karolinska Institutet, Stockholm, Sweden

**Keywords:** imagination, imagery neural systems, functional module, volume transmission, wiring transmission, modifiers, astrocyte networks, exaptation/mis-exaptation

## Abstract

This work aims at presenting some hypotheses about the potential neurobiological substrate of imagery and imagination. For the present purposes, we will define imagery as the production of mental images associated with previous percepts, and imagination as the faculty of forming mental images of a novel character relating to something that has never been actually experienced by the subject but at a great extent emerges from his inner world. The two processes appear intimately related and imagery can arguably be considered as one of the main components of imagination. In this proposal, we argue that exaptation and redeployment, two basic concepts capturing important aspects of the evolution of biological structures and functions (Anderson, 2007), could also be useful in explaining imagery and imagination. As far as imagery is concerned it is proposed that neural structures originally implicated in performing certain functions, e.g., motor actions, can be reused for the imagery of the virtual execution of that function. As far as imagination is concerned we speculate that it can be the result of a “tinkering” that combines and modifies stored perceptual information and concepts leading to the creation of novel “mental objects” that are shaped by the subject peculiar inner world. Hence it is related to his self-awareness. The neurobiological substrate of the tinkering process could be found in a hierarchical model of the brain characterized by a multiplicity of functional modules (FMs) that can be assembled according to different spatial and temporal scales. Thus, it is surmised that a possible mechanism for the emergence of imagination could be represented by modulatory mechanisms controlling the perviousness of “modifiers” along the communication channels within and between FMs leading to their dynamically reassembling into novel configurations.

## Introduction

### General premises

The main topic of the present paper concerns the possible neurobiological substrate of imagery and imagination. Imagery and imagination are commonly conflated concepts and indeed the term imagination is often used to name the general faculty of image production (Thomas, 1999). Despite the two terms sharing obviously many semantic features, *imagination* usually indicates the faculty of creating mental images and constructs of a novel character, a capability that appears to be especially developed in humans. In this view, the more basic faculty of producing mental images of previously experienced material, i.e., *imagery*, provides elements necessary for imagination and is arguably present also in other species together with a primitive form of imagination, namely a rudimentary form of prospection, i.e., the faculty of creating a mental picture of a future or anticipated event.

For the present purposes, we will adopt the following provisional definition of the two terms, based on a standard language reference (Webster’s Dictionary, second edition, Prentice Hall Press, NY, 1972) and the extant scientific literature:

- Imagery: the production of explicit and implicit mental pictures or images. Implicit mental imagery occurs when perceptual information is accessed from memory (Kosslyn et al., 2001), giving rise to the experience of “seeing with the mind’s eye,” “hearing with the mind’s ear,” and so on. By contrast, perception (explicit mental image) occurs as a result of a form of energy impacting directly on the sensory surfaces of the organism, generally via a constructive process (see e.g., Thomas, 1999)^1^
- Imagination: the act or power of forming mental images of what is not actually present or has never been actually directly experienced. Notably, imagination not only has the potential to enrich the meaning of an experience and deepen understanding, by multiplying and expanding the perspectives from which a phenomenon can be considered, but it also allows anticipating the outcome of an action without actually performing it via a “simulation” process. At its peak, imagination is the very mental faculty underlying visionary and creative thought.

The distinctive feature of imagination, therefore, rests on its capacity of creating new mental images by combining and modifying stored perceptual information in novel ways and by inserting this information in a subjective view of the world: hence it is related to his self-awareness. In other words, imagination is not simply the organization, identification, and interpretation of sensory information in order to represent and understand the environment but rather a constructive process that builds on a repertoire of images, concepts, and autobiographical memories and leads to the creation (and continuous update) of a personal view of the world, which in turn provides the basis for interpreting future information. Summing up it could be proposed that imagination represents the ability by the human subjects of creating novel “mental objects” that are shaped by their own peculiar inner world^2^. Given the above general considerations, we would like now to turn more specifically to neurobiology with the following distinction:

- Imagery is the capability of neural circuits to implement/provide a representation of an object that is not currently present in the subject’s sensory environment, but which the subject has had previous experience of; as a limit case, a specific mental image may even originate from an innate, genetically-encoded engram. Imagery can trigger appropriate behavior as real environmental stimuli do. This capability is thought to be present in several mammal species especially in great apes. In humans, imagery has been investigated in the motor domain (see below), visual domain (Chatterjee and Southwood, 1995; Kosslyn et al., 2001), and auditory domain (Zatorre and Halpern, 1993; Kosslyn et al., 2001). A general finding of these studies is that the brain areas activated during perception and during imagery overlap to a large degree (Kosslyn et al., 2001).
- Imagination is the capability of neural circuits to combine in novel ways images with a direct perceptual origin and concepts to produce original images and speculations. Through imagination, we are able not only to anticipate future events to a certain degree, but also to enrich our sense of the world and even conjure up alternative worlds, such as utopias^3^. Imagination can also be strongly related to inner speech^4^, which is a feature of human self-awareness and of crucial importance for the critical analysis of the virtual scenarios provided by the simulation processes. In particular, simulation has been recognized as an important mean for our understanding of social perception. In fact, simulation can make it possible to know what it feels like to perform actions, by recruiting neural circuits that partially overlap with those activated during the execution of such actions (Jabbi et al., 2008; Rizzolatti and Fabbri-Destro, 2008). Simulation processes play an important role not only when we think about the upcoming actions of another person, but also when we think of our own future. Buckner and Carroll (2006) have suggested that envisioning the future (prospection), remembering the past, conceiving the viewpoint of others (theory of mind), and spatial navigation are all specific instances of a more general process of “self-projection,” which involve medial temporal, parietal, and frontal circuits that also appear to have a high metabolic activity during passive rest and have therefore been termed “default mode network” (DMN) (Raichle et al., 2001).

### Aims of the present paper

These introductory remarks provide a useful starting point to examine the neurobiological relationship between imagery and imagination. We propose that the concept of exaptation (Gould and Vrba, 1982), introduced in the field of evolution, can be extended to neurobiology and be of relevance to explain the emergence of imagination from imagery.

**Figure 1:**
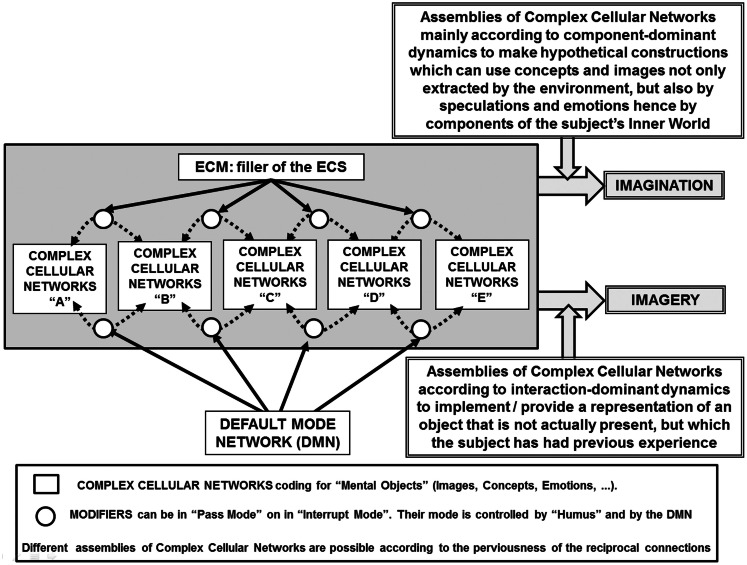
**Schematic representation of the main features of the present hypothesis on the combinatorial aspects that by assembling neural groups can lead to “imagery” and to “imagination.”** Details of the schema are briefly described in the figure and discussed in the paper. It is also indicated the extracellular matrix (ECM) as filler of the extracellular space (ECS) that can have a role in modulating the assembling of neuronal groups and their intercellular interactions (Agnati et al., 2006).

A general scheme of the present hypothesis, and the main assumptions of our argument that will be discussed in detail below, can be summarized as follows (see also Figure 1):

- Some neuronal systems can have two capabilities: to perform a function and to create in the internal theater of the subject (Baars et al., 2003) the virtual performance by the subject of that function. Clear examples are “mirror neurons” (Rizzolatti and Fabbri-Destro, 2010) and “motor imagery” (MI) (Decety and Ingvar, 1990; Decety and Grézes, 1999; Fleming et al., 2010). The link between the well-documented properties of mirror neurons and MI will be examined for its usefulness in shedding some light on the largely unexplored field of the neurobiological substrate of imagery.
  Thus, the possible existence of “imagery neuron systems” (INS) can be suggested and understood in the context of the redeployment hypothesis and of the interaction-dominant dynamics (Anderson et al., 2012). These concepts were proposed by Anderson and collaborators to classify the types of dynamics occurring among components of a system. In component-dominant dynamics, behavior is the product of a rigidly delineated architecture of modules, each with predetermined functions. On the contrary, interaction-dominant dynamics is crucially based on the plasticity of the system components and of the communication modes among these components. Anderson and collaborators maintain that the brain can be better described as an interaction-dominant dynamics system where coordinated processes alter the integrative action of components and where it is difficult, and sometimes impossible, to assign tightly defined and unique roles to specific components.
- On this assumption we propose that an exaptation phenomenon (Gould and Vrba, 1982) may have occurred during evolution favoring the emergence of imagination from imagery in a neurobiological context capable of self-awareness.
- From a more specific neurobiological point of view a key characteristics of these neuron systems that allow exaptation and redeployment is their dynamical organization into functional modules (FM). In the present paper and in previous articles (see Agnati and Fuxe, 1984; Agnati et al., 2012a) we have proposed that FM are organized according to a Russian Doll pattern (Jacob, 1970, 1977) where networks of different scale are nested within each other from cell circuit level down to the molecular level. Notably, FMs have no anatomically well-defined boundaries but rather represent computational units where transiently assembled, close-by structures cooperate in elaborating information and yielding an integrated response. It should be noted that the three-dimensional organization of FMs allows a huge number of combinatorial reassembling of the computational units encased at the different miniaturization levels within each FM.
- The communication among and within FMs can occur via the two modes of intercellular communication in the brain, namely “wiring transmission” (WT) and “volume transmission” (VT) (see Box 1). By virtue of such communications, supra-cellular networks can be created as “mosaic assemblies of FMs” (Agnati et al., 2010a). An important role in this process is likely to be played by astrocytes and by the extracellular matrix (ECM), both in the structural and functional organization of FMs and in the assembly of the mosaics of various hierarchical orders.
  In the present paper, we will speculate on how the combination of these communication modes in the brain can lead to the emergence of imagination by dynamically modulating the composition of mosaic assemblies and intra-mosaic computational processes. The combinatorial assembling of FMs into innovative mosaics is made possible by the presence of “modifiers” (a concept borrowed from the pioneering work of Alan Turing, see Box 2) along the communication channels that can be made by synaptic contacts (chemical and/or electrical) or by extracellular fluid pathways in the brain where chemical signals can diffuse. Given the variety of tasks that the brain can perform, and particularly for the emergence of imagination in humans, a “combinatorial optimization” mechanism that is able to find the optimal assembly for a certain output from a finite set of brain circuits is probably in operation (Lawler, 2001) and may play a role also in prospection.
- On the basis of the above definition of imagination it is deduced that imagination is possible only if the subject is capable of a fully developed self-awareness (Hobson et al., 2006; Jackson et al., 2012). Several authors suggest that the so-called Von Economo neurons (VENs) are involved in the self-awareness (Evrard et al., 2012) hence it can be surmised that VENs play a role in imagination processes. In agreement with such a view alterations in VENs have been described in autistic patients (Santos et al., 2011) as well as an impoverishment of their imagination capabilities (Craig and Baron-Cohen, 1999; Low et al., 2009).

Box 1 **Intercellular communication in the brain has been previously proposed by us to occur according to the two main modes of volume transmission (VT) and wiring transmission (WT).**

Furthermore, since the integrative actions of the CNS depend on all cell types present in the CNS we have also introduced the concept of “complex cellular networks” (Agnati and Fuxe, 2000). A complex cellular network (CCN) can be defined as the set of cells of any type, which exchanges signals in a certain volume of brain tissue and, thanks to this cross-talk, is capable of integrating inputs to give appropriate outputs. Volume transmission (VT) and wiring transmission (WT) can be distinguished based on the communication channels connecting the signal source with the signal-target. In the case of cellular networks, VT is due to the three-dimensional propagation of the electrical and/or chemical signal in the extracellular space of the brain (as well as in the cerebrospinal fluid) for distances greater than the width of the synaptic cleft, by diffusion and/or convection. Wiring transmission is characterized by a structurally well-defined channel (a “wire”) connecting the source-node with the target-node, i.e., it is realized by the physical contact (gap junctions) or quasi-contact (chemical synapses or membrane juxtapositions) between cells of the CCN. A high “transmission privacy,” usually present, due to the fact that the channel is a private channel.

Since the extracellular space of the brain is filled up with the extracellular matrix (ECM), the composition and tightness of its three-dimensional meshes has the greatest relevance on VT and also on WT (Agnati et al., 2006).

Box 2 **In a fascinating and farsighted report written in 1948, Turing (1994) suggested that the infant human cortex could be seen as an instance of an “unorganized machine”.**

Turing defined the class of unorganized machines as largely random in their initial construction, but capable of being trained to perform particular tasks. There is good reason to consider the infant cortex “unorganized” in this sense: there is insufficient storage capacity in the DNA which controls the construction of the central nervous system to exactly specify the position and connectivity of every neuron and by not hard-wiring brain function before birth we are able to learn language and other socially important behaviors which carry great evolutionary advantage.

Turing gives two examples of artificial unorganized machines, which he claims are about the simplest possible models of the nervous system: the Type A-machines and the Type B-machines.

The present paper considers the Type B-machines that can be described as a network where the perviousness of the inter-node connections are under the control of a connection modifier which can be in two opposite states: the pass mode or the interrupt mode. The connection modifiers greatly facilitate training by an external agent by allowing functional modifications to be made at any point in the network.

Turing also discussed input, output, and training structures for B-type machines and the possible role of “teaching signals” acting on the state of the modifiers (Turing, 1994). Turing mainly considered external teaching signals to be used for training the network function, but he also discussed networks capable of self-modification. As most connection modifier settings do not result in useful network behavior, Turing intended training to involve a systematic process where connection modifier settings are changed until the network is able perform a desired task. A set of useful modifier settings would constitute a kind of fixed program for the network. Thus, by way of such training and functional manipulation and via redeployment of the interconnected neural groups, multiple networks will be transiently formed and, even when the mosaics forming the network contain a limited number of tesserae, a very large repertoire of outputs would be possible.

It is clear that some assumptions of our argument have experimental evidence, while some others are only testable hypothesis or even speculations.

## The “Imagery Neuron Systems”

**Figure 2:**
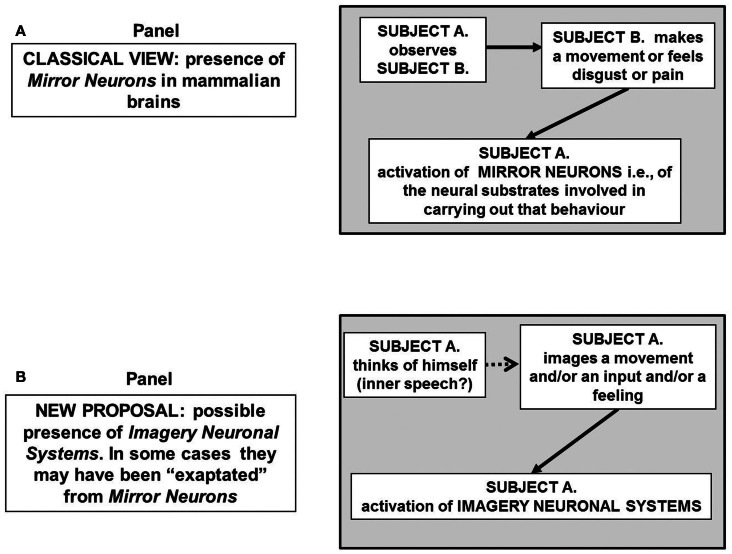
**Schematic representation of the possible block diagram representing the functional links leading to the “mirror neurons” that is of neurons that neuron “mirror” the behavior of activated motor neurons even if the observer is not really acting (A) hence mirror neurons sub serve two different functions**. In **(B)** the hypothesis is put forward that some mirror neurons could be part of a broader system, the imagery neuronal system, existing in the human brain, and likely in the brain of at least great apes. The activation of such a system is not necessarily dependent on a sensory input. For further details, see text.

A cognitive task of particular interest for the present discussion is that carried out by the so-called “mirror neurons.” As discovered and investigated in several important papers by Rizzolatti’s group, mirror neurons fire both when an animal acts and when the animal observes the same action performed by another subject (Rizzolatti and Craighero, 2004). Thus, the neuron “mirrors” the behavior of an activated motor neuron even if the observer is not really acting (Figure 2A) hence it does sub serve two different functions. Brain activity consistent with that of mirror neurons has been found in the premotor cortex and the inferior parietal cortex in humans (Rizzolatti et al., 2006; Ferrari et al., 2009). Such neurons have been observed in primates, and are believed to occur in other species including birds. Functional magnetic resonance imaging (fMRI) experiments suggest that rather than mirror neurons the human brain areas have mirror neuron systems (Iacoboni, 2009). As a matter of fact, fMRI studies in humans suggest that a much wider network of brain areas shows mirror properties than was previously thought (Gazzola and Keysers, 2009).

As pointed out by Rizzolatti et al. (2009), the mirror mechanism is located not only in centers that mediate voluntary movements, but also in cortical areas that mediate visceromotor, emotion-related behaviors. For instance, when a subject observes emotions in others caused by disgusting stimuli or stimuli representing pain, the cingulate cortex and the insula are activated; notably, the same areas are activated also when the subject herself experiences pain or disgust.

In other words, the anterior insula and adjacent frontal operculum (IFO) activate when individuals view or become aware of the delight, pain, or disgust of others, as when they experience these emotions first-hand, and this activation is modulated by individual empathic tendencies. In agreement with these findings, IFO lesions disrupt experience and recognition of disgust, suggesting a role for this region in emotional simulation/understanding (Jabbi et al., 2008). Both feeling emotions and recognizing emotions in others appear to be mediated by an integrative mechanism involving at least at a certain extent a common neural substrate.

In other words, the translation of a second-person observed behavior into a meaningful first-person percept employs to a great extent the same neural substrates which are involved in carrying out that behavior. Extant data (see Molenberghs et al., 2012) suggest that this holds true for motor behavior, visual, and auditory perception as well as for emotions.

These findings provide the basis for a more general hypothesis suggesting the existence of INS, that is, of neural systems capable of making representations of objects and feelings of which the subject has no direct experience. It may also be surmised (see Figure 2B) that mirror neurons are part of such a broader system existing in the human brain and likely in the brain of at least great apes. Furthermore, it could be proposed that mirror systems and more generally INS, by reflecting the relevant cues of the environment use an “analog code” that is the most useful code for the task the brain should tackle in that instance, e.g., a motor code in the case of a movement, an emotional code in the case of a feeling.

It is worth noting that this hypothesis finds consistency with two basic concepts that were proposed to capture significant aspects of the evolution of biological structures and functions: exaptation and redeployment.

The concept of exaptation has been introduced by Gould and Vrba (1982) pointing out the different role in evolution played by exaptation versus adaptation. While adaptation refers to a feature produced by natural selection for its current function, exaptation has been defined as a feature that performs a function but that was not produced by natural selection for its current use (such as feathers that might have originally arisen in the context of selection for insulation and not for flying; see, e.g., Xu et al., 2012). However, natural selection may subsequently operate upon such a new function to better adapt it to the possible new environmental requests.

In a somewhat similar theoretical frame Anderson has introduced the concept of evolutionary redeployment (or reuse) of a neural structure for a new function (Anderson, 2007, 2010). There are obvious advantages of redeployment of brain areas and Anderson analyses this phenomenon in relation with cognition.

From a general point of view it should be pointed out that the acquisition of the capability of performing a new function by a structure previously developed to carry out another function can lead to two different possible cases:

- The acquisition of the capability of performing the new function is accompanied by the loss of the capability to perform the original function;
- The acquisition of the capability of performing new functions is accompanied by the preservation of the capability to perform the original function. This case is probably better described by the term “multiple redeployment” of the structure (Anderson, 2010). In this context Anderson, by a careful survey of neuroimaging data, has pointed out that, for example, the Broca area is involved in multiple functional cognitive tasks besides containing motor neurons involved in the control of speech (Grodzinsky and Santi, 2008; Anderson, 2010).

Thus, the imagery capabilities can be described, according to these theoretical frameworks, as a sort of redeployment process, in which neural structures that are engaged in some action are also reused for the imagery of events involving that action.

This raises a further question: which neurobiological substrates could make possible such redeployment processes?

## The Three-Dimensional Organization of Brain Networks

**Figure 3:**
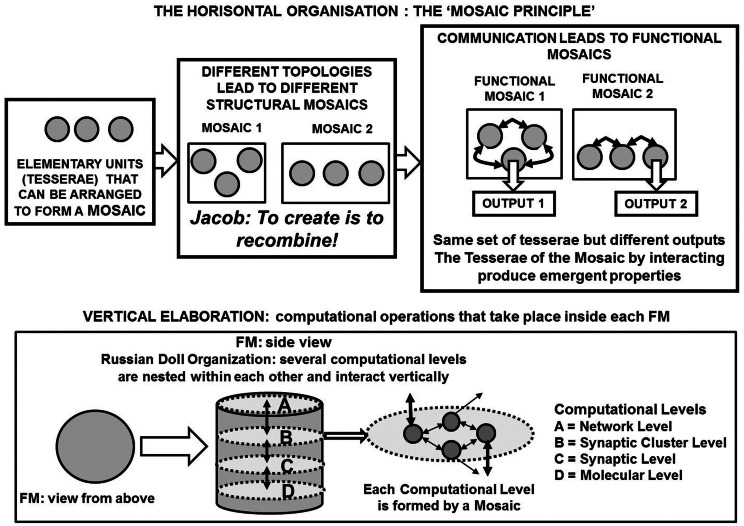
**A schematic representation of the functional module (FM) as a basic structure of the Russian Doll organization of the central nervous system (CNS)**. The three-dimensional elaboration of the information inside each FM is pointed out by remarking the horizontal elaboration that takes place within a hierarchic (miniaturization) level and the vertical elaboration that takes place among the various hierarchic (miniaturization) levels.

As briefly mentioned above, it has been proposed that the CNS can be described according to two main organizational paradigms:

(1) The horizontal mosaic organization, that is, the more or less stable assembly of structures, see Figure 3 (upper panel) in mosaics capable of emergent properties (Jacob, 1977). It should be noted that the concept of mosaic highlights the importance of topology (spatial location of the tesserae), however the temporal pattern, i.e., the *order of activation* of the tesserae of the mosaic, may also be crucial.
(2) The vertical (Russian Doll) organization (Jacob, 1970) of the FMs [Figure 3 (lower panel)] (see above and Agnati and Fuxe, 1984; Agnati et al., 2007, 2012a; Guidolin et al., 2011). It can be surmised that inside each FM there is a mosaic carrying out a crucial function and this mosaic likely is formed by synaptic clusters (SCs). Available data demonstrate that a gradient of communication processes transiently originates inside the SCs, which can recruit other structures (Shepherd, 1979; Golding et al., 2002). Thus, each FM is a computational unit only loosely delimited in functional terms, which sends integrated outputs to other FMs. Further details of such a paradigm are given below and have been discussed elsewhere (Agnati et al., 2012a).

Let us briefly discuss in more detail the neurobiological mechanisms capable of affecting FM boundaries, mosaic assembly and intra-mosaic dynamics.

As far as the FM boundaries are concerned, it can be argued that in mammalian brain the astroglial cells define the micro-architecture of the parenchyma by dividing the gray matter [through the process known as “tiling” (Bushong et al., 2004)] into relatively independent structural units. In particular, the protoplasmic astrocytes create micro-anatomical domains within the limits of their processes that can play a role in organizing SC, given that within these anatomical domains astrocytes isolate synapses and send their processes to plaster the wall of the neighboring blood vessels (Verkhratsky et al., 2011). Thus, astrocytes seem to have a basic role in shaping FM structures and functions, especially by affecting the possibility of SCs assembling.

Synaptic clusters are likely at the core of a FM by being a structural and functional bridge between the cellular networks and the molecular networks. It should be noticed that SCs have been shown to be very plastic entities from both the structural (Holtmaat et al., 2005) and the functional (Welzel et al., 2010) point of view. Notably, it has been reported that plastic changes induced by long term potentiation (LTP) at one synaptic contact lowers the threshold for the induction of LTP at neighboring synapses at a stimulation strength that did not cause any plastic changes under control conditions (Golding et al., 2002).

The extent of this sensitized plasticity zone spans about 10 μm of the dendritic tree and lasts for 10 min affecting neighboring spines (Harvey and Svoboda, 2007). These characteristics allow the SC to act as a sort of “intelligent layer” between the activity at cellular level and the integrative functions performed at molecular level by supra-molecular complexes such as those formed at the cell membrane by G protein-coupled receptors, owing to direct receptor-receptor interactions (see Fuxe et al., 2007; Agnati et al., 2010b; Kenakin et al., 2010; for reviews).

The relevance of SCs can also be deduced by the possibility of reverberating micro-circuits formed by the synaptic contacts belonging to a SC. As pointed out in a previous paper (Agnati et al., 2012a) a crucial quantitative difference in neuronal density (neurons/mm^3^ in layers I–VI) distinguishes the human brain from other species. Neuronal density in the cerebral cortex is lower in humans (24186/mm^3^) than in rats (54483/mm^3^) and mice (120315/mm^3^), whereas the number of synapses per neuron is higher in humans (29807) than in rats (18018) and mice (21133) (DeFelipe et al., 2002). The number of dendritic spines of basal dendrites of layer III pyramidal neurons also differs in mouse and human temporal cortex. The mean number (mean ± SEM) of spines per 10 μm segment is 10.9 ± 0.5 for cells in temporal cortex of mice and 14.2 ± 0.4 with respect to the temporal cortex of humans (Benavides-Piccione et al., 2002).

It should be noticed that a high density of synaptic contacts per neuron allows several alternative pathways in the high density local circuits of the human brain, and therefore a recirculation of the information that may be of fundamental importance for keeping information available for further integrations (DeFelipe et al., 2002; Alonso-Nanclares et al., 2008), including those implicated in memory processes (Agnati et al., 1981; Douglas et al., 1995; Goldman-Rakic, 1995; Romo et al., 1999; Wang, 2001).

Functional modules in the brain could be assembled via both WT and VT (see Agnati et al., 2010a) to form mosaics capable of highly integrated actions. Furthermore, individual astroglial domains have been hypothesized to be integrated into the superstructure of astroglial syncytia through gap junctions localized on the peripheral processes of astroglial cells (Verkhratsky et al., 2011), hence potentially playing a fundamental role in the assembly of FM mosaics. Actually, Pereira and Furlan (2010) have proposed the existence of an active astroglial network functioning as a “master hub” that integrates elaborations carried out by distributed brain areas and supports conscious states. Following Kelso (2009) FMs could be characterized as “synergies.” “Synergies” are systems exhibiting interaction-dominant dynamics; more specifically, a synergy is a functional grouping of structural elements (molecules, genes, neurons, muscles, limbs, individuals, etc.) that are temporarily constrained to act as a single coherent unit (Kelso, 2009). In this perspective, each FM is a synergy (since its response is the integration of the elaborations carried out at multiple scales) and synergies are also the mosaics of FMs (see below and Agnati et al., 2012a).

In this context a further organizational aspect deserving consideration concerns the possibility (see Agnati and Fuxe, 2000) that the communication pathway between a signal source and its targets could be under the control of a “modifier,” responding to teaching signals that could originate, for instance, from environmental inputs. Such a modifier could be spatially situated along a VT diffusion pathway and/or an astrocyte network connecting different FMs (Figure 1). It should be noted that this logical scheme, in a loose way, recalls a feature of the Turing B-machine (see Box 2 and Turing, 1994; Agnati and Fuxe, 2000; Guidolin et al., 2007).

The modifier can be simply a suitable arrangement of glial cells that can open (pass mode) or close (interrupt mode) the extracellular space pathway via a differential swelling and thus control slow VT communications between the source of a signal and its possible targets (Agnati and Fuxe, 2000). Suitable signals could also affect gap-junction connections between astrocytes (Giaume, 2010) and therefore the fast transmission of the information along this channel. The existence of such modifiers could be of the greatest importance for the information transfer through a master hub integrating such information by means of calcium waves prompted by synergic factors and propagated or not according to the opening and closing of astroglia gap junctions. It should be noted how this proposal is basically in agreement with our suggestion of the possible existence of modifiers along the communication channels between the tesserae of mosaics and hence the potential role of teaching signals addressing the flow of information.

Altogether these structural and functional features could allow a huge combinatorial possibility for the realization of different computational assemblies (mosaics) between and within FMs, leading to the extraordinary variety of the brain integrative actions (see Agnati et al., 2012a). Different higher-order mosaics can be transiently assembled by the same set of FMs, hence a multi-redeployment process of the same brain areas can occur, allowing the execution of markedly different brain integrative actions. In the proposed FM mosaics framework, a key concept is that of “reuse” of the same tesserae also within the FMs to build many different mosaics capable of different patterns of activity leading to different integrative actions. Thus, the just described organization can provide a suitable substrate to the Anderson’s proposal of the creative reuse of existing neural components (see above), a concept which is also in line with the “tinkering principle,” proposed more than three decades ago by Jacob (1977) who stated that “evolution tinkers together contraptions and novelties come from previously unseen associations of already available material” (see Figure 3 upper panel).

In this respect, the present view not only proposes a neurobiological basis for some general cognitive theories appeared in the last 5 years (Gallese, 2008; Hurley, 2008; Dehaene, 2009; Anderson, 2010) suggesting that low-level neural circuits are used and reused for various purposes in different cognitive and task domains, but is also in line, to some extent, with Hebb’s classical hypothesis on the possible existence of reverberating circuits interconnecting cell assemblies (Hebb, 1949). It also shows consistency with the important concept of “neural re-entry” introduced by Edelman (1999). Such a concept indicates a process which coordinates neural maps through the parallel selection and correlation of neuronal groups in different areas of the brain and makes possible the emergence of complex sensory and cognitive features. Thus, Edelman points out that we ought to regard a complex system as one in which smaller parts are functionally segregated or differentiated across a diversity of functions but also as one that shows increasing degrees of integration when more and more of its parts interact.

The main difference with the abovementioned theories is that our proposal maintains that brain integrative actions are achieved by means of communication processes among and within FMs allowing a simultaneous “three-dimensional elaboration of the information” (Figure 3 lower panel). According to the present proposal of the CNS organization, for instance, it can be surmised that neural re-entry could also occur within each FM, between the networks of different miniaturization composing the FM itself.

In summary, we propose that redeployment processes could occur at multiple spatial (and temporal) scales within and between FMs belonging to brain regions not necessarily close to each other anatomically, when cooperating in a cognitive task. This means that a combinatorial interaction-dominant dynamics is in operation and this process is crucially based on the plasticity of the system components (nodes and channels). As a consequence, different combinatorial assemblies of FMs can occur via the appropriate resetting of the modifiers allowing the performance of different functions by a restrict numbers of neural groups. Such a combinatorial reuse of the plasticity of the connections and of the computational nodes allows the tuning of responses and may even lead to the emergence of imagery and imagination in humans and hence of a subjective fantastic realm that is one of the most important source of creativity^5^ (see Figure 1).

## On the Possible Role of Interaction-Dominant Dynamics in the Emergence of Imagination in Humans

The above illustrated concepts could also be applied to the emergence of imagination, starting from the evidence of the existence of INS, and particularly from the well-characterized case of MI (see Munzert et al., 2009, for a review). MI can be defined as a dynamic state during which an individual mentally simulates a given action. This type of phenomenal experience implies that the subject feels herself/himself performing the action (Decety and Ingvar, 1990).

Thus, MI has been shown to involve the conscious internal representation of movement, without overt motor performance (Decety and Grézes, 1999; Fleming et al., 2010). Similarities in brain activation between MI and actual movement have also been demonstrated by fMRI (Gerardin et al., 2000). Actually, during imagined and executed finger movements, common areas of brain activation can be observed in the premotor cortex, supplementary motor area, and parietal cortex, with activation peaks slightly more rostral in frontal areas and slightly more superior and caudal in parietal areas during MI compared with movement execution (Gerardin et al., 2000).

An overlap between executed, observed, and imagined reaching activations was found in dorsal premotor cortex as well as in the superior parietal lobe and the intraparietal sulcus (Filimon et al., 2007), thus providing evidence for at least a partial overlap between the neural circuits activated by MI and the mirror neuron systems.

It is interesting to underline an important functional finding that has been not yet explained namely that both the right and left parietal cortices show greater blood oxygen level-dependent signals during imagery than during motor execution (Fusi et al., 2005).

In addition, the greater activation of the superior parietal cortex during imagery than during preparation for movement, indicates that MI is more than simply readiness to move (Stephan et al., 1995). These results give further indications of the importance of imagery processes and of the possible existence of INS that may be in part overlapping mirror neurons systems, but may also be independent from them not only as far as motor behavior but also emotions are concerned (see below the possible key role of VENs).

All these findings on INS can suggest that the emergence of imagination in humans may be the result of two processes:

- Exaptation and/or redeployment of a peculiar type of FMs where some type of INS plays a basic role in the integrative action of those FMs;
- A peculiar interaction-dominant dynamics triggered by VENs leading to the assembly of FM mosaics where FMs containing INS have a hub role.

We propose that the activation of the INS and the assembly of mosaics of FMs containing this type of neuronal systems may be a prerogative of the DMN (Raichle et al., 2001).

Thus, we propose that in the human brain specialized INS, possibly VENs, under the control of the DMN may provide the neurobiological substrate for an effective massive redeployment (Anderson, 2007) of local networks to many different higher-level cognitive tasks such as imagery, prospection, and imagination.

In particular, we will discuss below whether INS can be part of mosaics of FMs, which thanks to an interaction-dominant dynamics and under the “creative” influence of the DMN form special mosaics from which “imagination processes” emerge.

### Possible reuse of imagery neurons for imagination processes

There is evidence that some mirror neurons are reused as INS and that this reuse holds true also for emotional behavior. However, it can also be hypothesized that in some instances an exaptated process could have taken place, leading to the peculiar characteristics of the imagery function. In this respect an important aspect to consider concerns the process used by the brain to acquire knowledge about the world. As pointed out by Zeki (2004), such a process is basically a diffuse process of abstraction, in which each FM has an abstractive machinery which transforms an incoming fragment of sensation into a particular fragment of perception, i.e., extracts a specific attribute of a sensory modality. Mechanisms of binding then allow these different components to be gathered into one global picture, generating general models of the external world (Blakemore, 1976). This process of generalization is a very efficient way in which a finite mind grasps the infinity of particulars, and also frees the brain from total dependence upon a memory system (Zeki, 2004). It could be surmised that the development of this ability could be the background on which the emergence of the typical human capability of imagination could arise.

On the basis of the definitions of imagery and imagination given in the introduction, it seems likely that the latter has emerged from the former one. In fact, imagination is the capability of the mind to create a hypothetical construct with concepts and images originated from the environment (i.e., provided by imagery), but also to fashion entirely new mental objects.

This assumption can be of some importance in showing a sort of continuity between a more basic function provided by mirror neurons, a higher-order one provided by INS (see e.g., MI), and an even more advanced one leading to imagination processes. These subsequent evolutionary achievements could have had a high social value by favoring intra-individual empathy.

In agreement with Bloch’s fascinating proposal (Bloch, 2008), also religious beliefs can be discussed under this perspective since imagination allows humans to deal with things and beings that do not exist physically. Thus, exclusively humans could use what Bloch calls the “transcendental social” to unite with groups, such as nations and clans, or even with imaginary groups such as departed ones and gods. In Bloch’s view, the transcendental social also permits humans to follow the idealized codes of conduct associated with religion; notably, the very existence of the transcendental social presupposes the capacity to assign a pregnant, “lived” meaning to the constructs provided by imagination.

Is it possible to propose a neurobiological substrate for imagination processes? The demonstration of MI in humans points to the possible activation of FM mosaics by an internal input that could or could not be triggered by an external input (see Figure 2B). For example, Dagher et al. (2008) demonstrated that abstract planning can activate motor areas even when the task to be planned involves no motor activity by itself.

The exaptation of auditory INS may also have led to the emergence in humans of the “inner speech.” This process is usually characterized by a semi-constant, involuntary, and non-articulated stream of verbal flow of associative thoughts that is perceived at a conscious or semi-conscious level of mental awareness (Morin and Everett, 1990; Morin, 2009); it could be argued that imagination plays a fundamental role for the inner speech, which certainly plays a role for predictive psychic homeostasis that is strictly linked to the subject prospection (Agnati et al., 2011). In the perspective outlined in the present work, inner speech may rely on a special class of auditory INS exaptated for sub-vocal verbal and imagination processes. If such a proposal can be investigated and proved it will allow a better understanding of the human consciousness and possibly also of some psychopathologies such as schizophrenia and autism.

Thus, it should be considered the possibility of a “mis-exaptation” of imagination processes leading in the most clinically relevant cases to intrinsic fuzziness and imprecision of brain function. This aspect has been discussed in a previous paper where the possible functional and genetic links between schizophrenia and creativity have been pointed out (Agnati et al., 2012b). Thus, in some cases creativity may reflect not so much the functioning of the brain but rather its borderline dysfunction hence when things go a little wrong, that something new is experienced^6^.

As mentioned above, a special class of neurons, known as VENs, could have an important role in imagination processes since they appear to be involved in the cognitive process of abstraction. In fact, only animals whose brains possess VENs pass the “mirror test” of self-awareness (Gallup, 1970), which aims to ascertain whether the animal possesses the ability to recognize himself in a mirror.

It has already pointed out that it is reasonable to assume that self-awareness is a necessary platform for imagination processes and therefore that exaptated VENs may be involved in such high-level cognitive function. As a matter of fact, some previously cited papers (see Introduction) report findings obtained in autistic patients that support a link between alterations in VENs and deficits in imagination processes. Thus, let us analyze the possible role of VENs in this faculty.

### On the neurobiological exaptation of imagery: Possible role of Von Economo Neurons

Von Economo neurons have been proposed to represent a necessary neuronal adaptation in very large brains (especially chimpanzees and humans), permitting long-range fast information transfer and integration along highly specific projections, and that evolved in relation to emerging social behaviors (Allman et al., 2005; Premack, 2007; Butti et al., 2009). In the hominid brain they are located in layer 5 of the anterior cingulate cortex (ACC) and the fronto-insular cortex (FIC) (Seeley et al., 2012; Cauda et al., 2013). In humans they have also been found in the dorso-lateral prefrontal and in the frontal cortex (Fajardo et al., 2008). The VENs have a narrow dendritic arborization that spans the layers of the cortex and may be able to sample and rapidly relay the output from a columnar array of neurons. The apical and basal dendrites of the VENs are remarkably symmetrical; this architecture suggests that the VENs may be comparing inputs from these two symmetrical dendrites.

The cell bodies of VENs in ACC are, on average, 4.6 times larger than that of layer 5 pyramidal cells in this area (Nimchinsky et al., 1999). The VENs’ large size suggests that they bear large, rapidly conducting axons, which is a characteristic feature of large neurons in layer 5 elsewhere in the cerebral cortex (Sherwood et al., 2003). Concerning comparative anatomy, Premack (2007) cites evidence showing that our species has not only many more and larger VENs than apes, but also far wider perimeters of dendrites (an average of 51 μm in humans compared with 36 μm in chimpanzees and monkeys).

The dendritic architecture of neurons reflects the way in which they integrate information (Vetter et al., 2001). Both spines (Sabatini et al., 2001), and branches (Polsky et al., 2004) can operate as processing compartments, and, compared with their layer 5 pyramidal counterparts, VENs have fewer of both, which suggests that the VENs receive fewer, but likely already integrated, inputs than pyramidal neurons. Thus, the radially polarized structure of the VENs could be a specialization for rapid signal transmission, providing an output that reflects the elaboration of the few highly relevant integrated inputs from the neurons and astrocytes of the FMs present in the ACC and FI to FMs of other brain regions.

Allman and collaborators tested adult human ACC and FI tissue for reactivity to antibodies raised against the vasopressin and oxytocin receptors. These authors have demonstrated that antibodies specific for the Vasopressin V1a receptor label a subpopulation of VENs, as well as pyramidal neurons in layers 2/3 and 5 of ACC and FI. The pattern of labeling was interesting since apical dendrites labeled with the V1a receptor antibody formed columns that spanned layer 5 to layer 1 (Allman et al., 2005, 2011). It has also been shown that VENs are strongly labeled with antibodies to 5-HT2B receptor (see below) and to the dopamine D3 receptor, which is a high affinity dopamine receptor that has been proposed to signal the expectation of reward under uncertainty (Le Foll et al., 2002).

In summary, it could be argued that VENs are a special type of “hub neurons” of FMs in the FI and ACC, which are capable of integrating a selected few WT inputs and several VT signals (also broadcasted via the CSF channel). Thus, VENs may carry out an extensive integration of multiple signals that can be rapidly communicated via their large axons to distant brain areas (Stimpson et al., 2011). This special role of VENs can also be deduced by the above mentioned observation that human VENs are located in only two parts of the human brain, the ACC and the FI cortex, which have been implicated in socially crucial processes, such as empathy, feelings of guilt, and embarrassment. In agreement with such a view, neuropathological data demonstrate that the selective destruction of VENs, as in the early stages of fronto-temporal dementia, is associated to profound deficits in empathy, social awareness, and self-control (Seeley et al., 2006).

The larger number of VENs in the right hemisphere may be related to asymmetry in the central control of the autonomic nervous system in which the right hemisphere is preferentially involved in sympathetic activation, resulting from negative feedback and subsequent error-correcting behavior; the left hemisphere is preferentially involved in parasympathetic activity associated with reduced tension or calming responses.

It may therefore be speculated that VEN predominance in the right hemisphere in the postnatal period is related to the right hemisphere specialization in humans, at least to the social emotions that are of basic importance for self-consciousness (Allman et al., 2005, 2011).

Furthermore, via their large, rapidly conducting axons VENs may rapidly relay this information to other parts of the brain and hence play an important role in the assembly of the mosaics of FMs involved in the imagery processes.

### Hypothesis on the neurobiological mechanism allowing the emergence of imagination

Analysis of fMRI-based resting-state functional connectivity, using both model-based and model-free approaches, has suggested the existence of at least three large-scale networks in the human brain (Sridharan et al., 2008):

- a central executive network (CEN), whose key nodes include the dorso-lateral prefrontal cortex (DLPFC), and posterior parietal cortex (PPC), regions routinely activated during goal-directed task performance;
- the DMN, which includes ventro-medial prefrontal cortex (VMPFC), posterior cingulate cortex (PCC), lateral parietal cortices, and the hippocampal/parahippocampal cortices. The DMN shows high metabolic activity and blood flow at rest but is commonly deactivated during a variety of externally directed tasks (Raichle et al., 2001);
- a salience network (SN), which includes the ventro-lateral prefrontal cortex (VLPFC) and anterior insula (jointly referred to as the FIC) and the ACC.

Thus, during the performance of cognitively demanding tasks, the CEN and SN typically show increases in activation whereas the DMN shows decreases in activation.

There is a general agreement that DMN is associated with stimulus-independent cognition (Buckner et al., 2008), however the debate continues on the specific role of the DMN. In fact, it is difficult to accept the view that such an extensive network with its elevated activity at rest is only due to spontaneous thought (Raichle and Snyder, 2007). It has been shown that the major nodes of the DMN are functionally and structurally connected and that this connectivity develops through ontogeny (Fair et al., 2008) and becomes relatively weaker both in the elderly (Andrews-Hanna et al., 2007; Damoiseaux et al., 2008), and in patients with attention-deficit disorder (Castellanos et al., 2008) and impulse control disorders (Church et al., 2009). The DMN has also been associated with phenomena such as self-referential processing, autobiographical recollection, mind-wandering, and theory of mind (Gusnard et al., 2001; Mason et al., 2007; Buckner et al., 2008).

**Figure 4:**
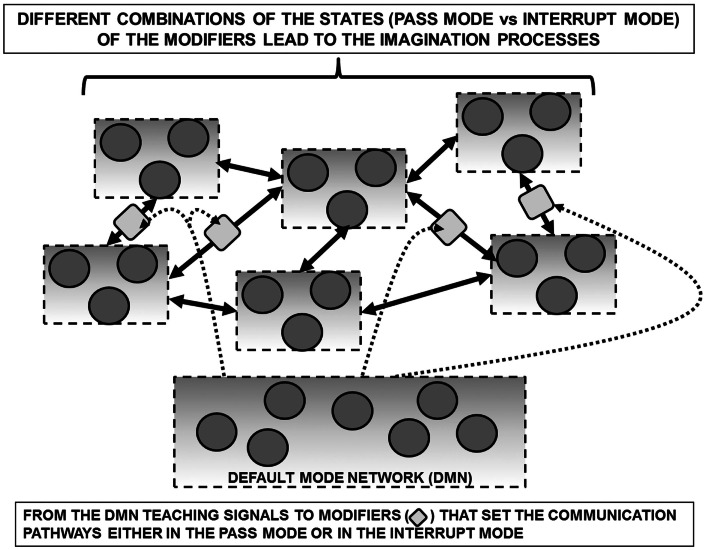
**Schematic representation of the default mode network (DMN) in a redeployment process via a suitable control on modifiers along communication pathways interconnecting FMs**. Such a process can be surmised allowing the assembly of FM mosaics that are not necessarily dependent from sensory inputs. In support of such a hypothesis is the observation that the DMN shows high metabolic activity and blood flow at rest but is commonly deactivated during a variety of externally directed tasks. For further details, see text.

**Figure 5:**
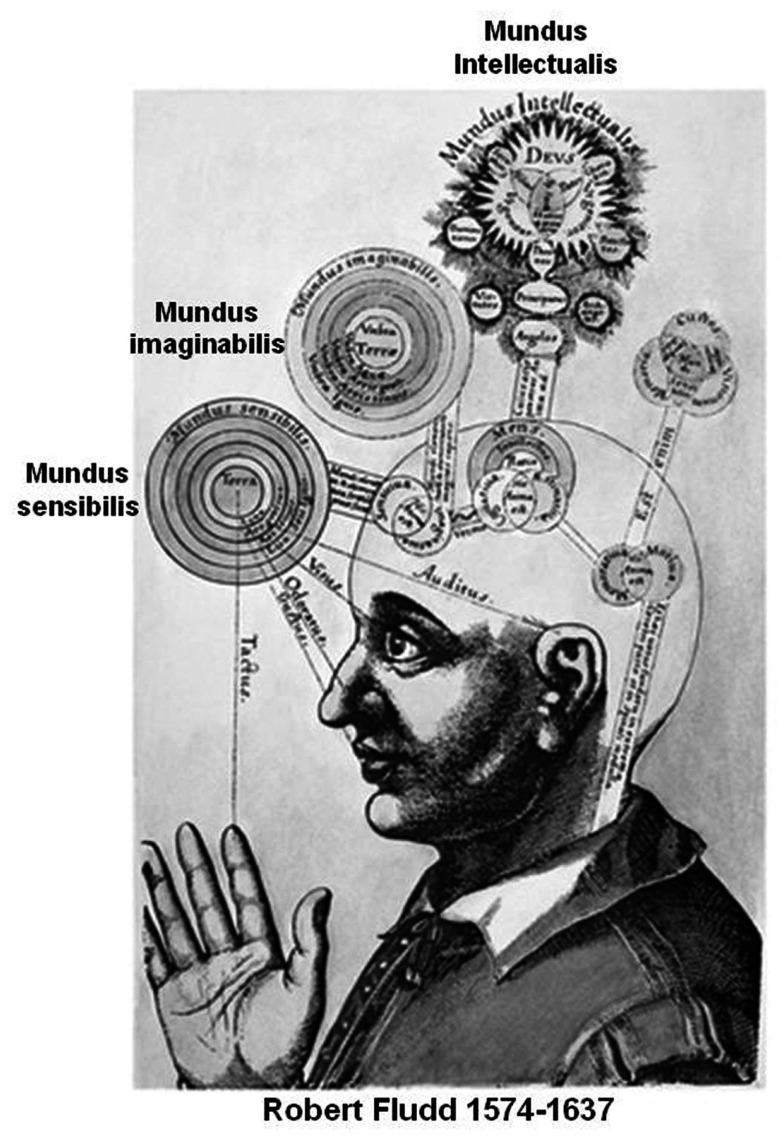
**Inspiring drawing from Robert Fludd masterpiece (*Utriusque Cosmi, maiores scilicet et minores, metaphysica, physica atque technica Historia* 1617–1621)**. In agreement with our proposal the imagination process can be considered as Fludd’s *mundus imaginabilis* hence a process that can be entirely decoupled from the input of the external world.

Based on these considerations, it can be hypothesized that the high activity of DMN during periods of unconstrained “rest” depends on the “creative” assembly of FM mosaics via the control of the modifiers along the pathways interconnecting single FMs as well FM mosaics into complex FM mosaics (Figure 4). Such a combinatorial process can be entirely decoupled from the inputs from the external world and can therefore correspond to the *mundus imaginabilis* proposed by Robert Fludd (*Utriusque Cosmi, maiores scilicet et minores, metaphysica, physica atque technica Historia* 1617–1621; see Figure 5).

**Figure 6:**
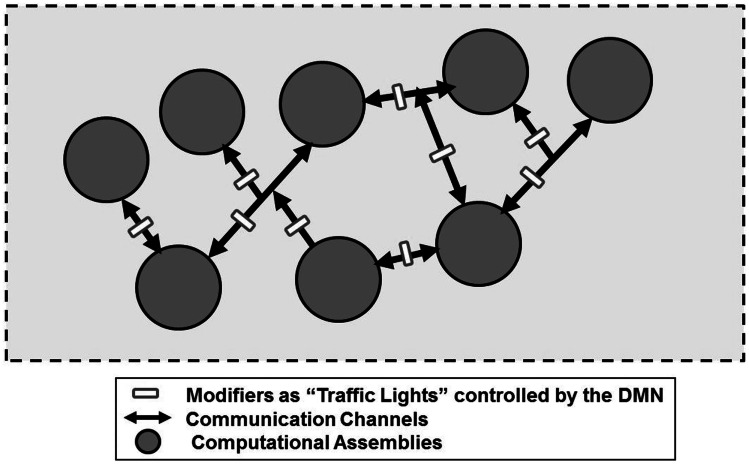
**Schematic representation of a new type of a hypothetical “machine” made by computational assemblies interconnected by pathways bearing “traffic lights” that can divert the signals along many different routes**. For further details, see text.

As schematized in Figure 6, it is even possible to surmise a new type of “machine” made by computational assemblies interconnected by pathways bearing “traffic lights” (modifiers) that can divert the signals along many different routes. The computational capability of this machine could be highly enhanced by a diffuse system of extracellular signals capable of affecting the ECM composition and/or geometry and hence controlling these traffic lights. An interesting question is whether such traffic lights are present, not only along the VT diffusion pathways and the Head Master network, but also at the input and/or output level of VENs, possibly as a pre-synaptic inhibition.

The putative crucial role of VENs is underscored by their localization in the anterior insular cortex (AIC) and ACC, which could be considered as input and output regions of a complex functional system crucially engaged for the production of subjective feelings and for coordinating appropriate responses to internal and external events (Medford and Critchley, 2010). In fact, AIC exchange information with ACC via VENs and, while AIC serves mainly to filter and relay autonomic and emotional information, ACC on the basis also of information from the prefrontal cortex is involved in the production of high-level voluntary responses. In line with the FM mosaic hypothesis, before triggering the final output this synergy of different brain areas builds up a variety of possible hypothetic scenarios and, among these, DMN is involved in selecting the final choice.

Summing up, imagination could emerge from many different peculiarities of the human brain, such as:

- redeployment of FMs containing possibly exaptated INS;
- especially developed connection-dominant dynamics allowing the assembly of a vast repertoire of FM mosaics;
- a peculiar role of astrocytes with a high supporting function allowing great plasticity of the components of the FMs especially SCs and, in general, favoring the assembling of a great variety of different FM mosaics, e.g., via their “tiling” function (Bushong et al., 2004).

**Figure 7:**
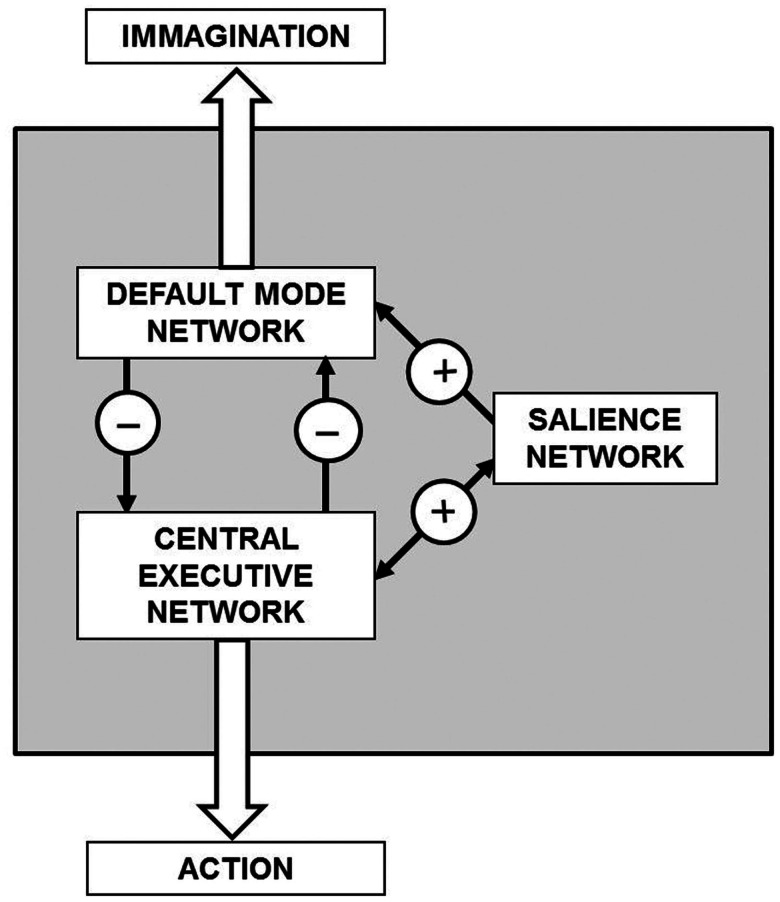
**General theoretical block diagram of the present hypothesis illustrating in a highly schematic way the cognitive control mechanisms that mediate concurrent activation and deactivation of three large-scale brain networks namely the default mode network, the central executive network, and the salience network during task performance or during imagination processes**. The detailed morphofunctional descriptions of these networks and how their integrative actions take place are still largely unknown.

A general theoretical scheme of the present hypothesis is illustrated in Figure 7. It should be noticed that remains unknown the crucial issue of the neuroanatomical and functional features of the control mechanisms that mediate concurrent activation and deactivation within these large-scale brain networks during task performance or during imagination processes.

## The Present Hypothesis in the Context of Available Theories of Human Imagery and Imagination

It is well known that many psychologists of the behaviorist era did deny the psychological significance of imagery. However, from the late 1950s interest in imagery gradually increased (Holt, 1964) and from the later 1960s a number of experimental findings (see Morris and Hampson, 1983) convinced many researchers of its cognitive significance, stimulating the development of theories on the nature and mechanism of imagery (see Pylyshyn, 2002). As pointed out by Thomas (1999), the extant theories proposed by cognitive sciences can be categorized according to three basic types that can be better understood as “research programs,” each characterized by certain explanatory strategies, but encompassing a number of diverse theoretical positions:

- The “quasi-pictorial” class of theories (Kosslyn, 1980) holds that quasi-pictures are constructed on the basis of sensory information in the form of “deep representations” and stored in functionally defined loci (like the files saved by a computer program). Once established, such an information can be extracted by a postulated “mind’s eye function.”
- The “description” theories (also known as “propositional” theories) share with the “quasi-pictorial” ones the idea that some type of data structure is a proper model for mental content. However, the data structures that they take to embody mental images (and percepts too) are regarded as expressing propositions descriptive (in some inner language) of the relevant perceptual scenes (Pylyshyn, 1991). When such structures are the end product of perceptual processes, we are perceiving; when they are constructed inventively, or retrieved from memory, we experience imagery.
- A different approach (not involving an information processing framework) is represented by the “perceptual activity” theories (Neisser, 1976) in which perceptual learning is not viewed as a matter of storing descriptions (or pictures) of perceived scenes or objects, but as the continual updating and refining of procedures (or “schemata,” see Neisser, 1976) that specify how to direct our attention most effectively in particular situations: how to efficiently examine and explore, and thus interpret, a scene or object of a certain type. Imagery is experienced when a “schema” that is not directly relevant to the exploration of the current environment is allowed to exert at least a partial control of the exploratory apparatus.

As extensively discussed by Thomas (1999) all these approaches face theoretical and empirical difficulties. The first two, in particular, implicitly rest on a view of the of the brain as a pure computational system or as a computational symbolic processor which does not appear to fully fit with the morphofunctional characteristics of the CNS as we know them today (Pani, 2002; Guidolin et al., 2011). Furthermore, the acknowledged relationship between imagery and imagination does not seem to be fully explained within these theoretical frameworks. The “perceptual activity” theories represent a field still under development, which, however, appears promising for a psychological theory of imagination (Thomas, 2009). In particular, they account for perception, imagery and imagination via a common mechanism (Thomas, 1999).

Unlike the abovementioned theoretical frameworks, the hypothesis here presented is mainly focused on neurobiological aspects. It suggests how a very general process (such as exaptation and redeployment) could drive the emergence of imagery and imagination capabilities starting from more elementary functions. In this respect, the hypothesis here presented shows some point of contact with “perceptual activity” theories. Moreover, the reuse of the same substrates for different task domains (perception, imagery, imagination) seems also in line with the classical view that “perception is a model in the brain” (Blakemore, 1976), meaning that it is not a passive response to sensory stimuli, but is rather an imaginative construction of possible perceptions which are then tested against the input. According to the hypothesis here presented, biological mechanisms making possible the process of redeployment could rest on a peculiar morphofunctional organization of the brain (see §2), in which communication processes among and within FMs allow the transient assembly of set of FMs opening the possibility to explore multiple processing schemes with the same basic structures.

Although a direct experimental demonstration or confutation of the present hypothesis is not straightforward, the abovementioned features can suggest possible lines of experimental testing. As far as the possible strong relationship between imagery and imagination is concerned, of great interest would be clinical and psychological investigations aimed at detecting the existence of complete non-imagers who are otherwise normally conscious and mentally competent. The question has been debated for more than 100 years (see Galton, 1880, 1883), but no systematic research whatsoever has been done on the phenomenon. Some recent paper (Faw, 1997, 2009) addressed the topic, but giving only very sketchy details of the methodology and the results.

Moving to more specific neurobiological aspects of the presented hypothesis, several experimental tests could be devised. Of particular interest, however, could be to find out procedures to evaluate the functional plasticity of the astrocyte networks, since they play an important role in the functional organization (and re-organization) of the FMs from specific interactions with single synaptic contacts (De Pittà et al., 2012) to modulatory interactions with entire neuronal networks (Schipke et al., 2008; Pereira and Furlan, 2010). In this respect, experimental studies designed to evaluate the amount of intra and extracellular factors (i.e., the “modifiers”) modulating of the gap-junctions in astrocyte networks (Franke, 2009; Giaume, 2010) and comparing this parameter between different species (e.g., rat and great apes) could provide some indication on the role of redeployment processes in the higher integrative functions of the CNS.

## Final Comment

It is worth noting that the present hypothesis suggesting some sort of combinatorial capability of neural and molecular circuits and the relevance of considering a three-dimensional elaboration of the information by the brain may also shed some light on a question that has been put more than one century ago: the importance of brain size for human cognitive capabilities.

In fact, it implies that, beyond a critical threshold, the brain size in different human beings is not a crucial parameter for their cognitive capabilities.

As a matter of fact, it has been pointed out in a very interesting article by DeFelipe (2011) that even a difference of almost 50% of brain mass in humans may have no functional significance in terms of intelligence.

Thus, not simply the increase in size but rather the complexity of brain circuits seems to be responsible for our higher, more abstract mental abilities. In particular, it can be surmised that of the highest relevance are the specializations of our cortical circuits made by complex cellular networks (i.e., mainly neurons, astrocytes, and ependymal cells) and the density and organization of SCs as well as the number and structure of the VENs.

In conclusion, cognitive capabilities are largely determined by the individual pattern of transient connections (i.e., the control of the modifiers state) that via a combinatorial process can form different FM mosaics. A further element of plasticity can be understood when the vertical elaboration of the information associated within each FM and its peculiarity, especially due to the integrative function of SCs, is considered.

## Conflict of Interest Statement

The authors declare that the research was conducted in the absence of any commercial or financial relationships that could be construed as a potential conflict of interest.

## Footnotes

^1^Rather than viewing perception as proceeding linearly from the inflow of sensory data into the brain, the perceptual activity theory (adopted, e.g., in *active vision* robotics), models it as a continual process of active interrogation of the environment: *impulses in the optic nerve fibers at each moment of scanning a scene are the answers, in code, to the “questions” that had been asked at the previous moment … [What] goes on in the retina is not like the recording of a “picture,” but the detection of a series of items, which are reported to the brain* (Young, 1978). Each new “question” takes its cue from the answers to the previous ones. See also the more recent formulation of a similar idea within a Bayesian framework in Friston (2005) and Friston et al. (2012).

^2^As an example, Romeo who thinks and sees with his mind’s eye Juliette kissing him would be an example of imagery, while the reader of the play who creates in his mind the scene of Romeo who thinks of Juliette’s kiss and thus enriches his inner world with such creation would be an example of imagination. The term*s fantasy and fantastic* (from Greek *phantázein, “*to present to the mind,” and *phantastikos*, “producing mental images”) are strictly related to the concept of imagination detailed here.

^3^The word comes from the Greek: *o*υ (”not”) and τ*ó*π*o*ς (“place”) and means “no place.” Utopia is an ideal community or society possessing a perfect socio-politico-legal system. The word was coined by Sir Thomas More for his 1516 book Utopia, describing a fictional island in the Atlantic Ocean.

^4^An extraordinary description of the links between inner speech and imagination can be read in Marcel Proust (Swann’s Way; Place-Names: The Name): *But if their names thus permanently absorbed the image that I had formed of these towns, it was only by transforming that image, by subordinating its reappearance in me to their own special laws; and in consequence of this they made it more beautiful, but at the same time more different from anything that the towns of Normandy or Tuscany could in reality be, and, by increasing the arbitrary delights of my imagination, aggravated the disenchantment that was in store for me when I set out upon my travels. They magnified the idea that I formed of certain points on the earth’s surface, making them more special, and in consequence more real. I did not then represent to myself towns, landscapes, historic buildings, as pictures more or less attractive, cut out here, and there of a substance that was common to them all, but looked on each of them as on an unknown thing, different from all the rest, a thing for which my soul was athirst, by the knowledge of which it would benefit*.

^5^It should be noted that an objective record of the fantastic realm of a subject’s, i.e., an important part of his inner world can be at least in part gathered by an observer if and only if these mental objects, *once translated in artworks, appear to the observer not simply bizarre but rich of a mysterious meaning* (see Caillois, 1965) and appears to be the result of a creative process. Actually, creativity has been defined as the capability of producing new contraptions that have a meaning or a utility and, hence, are not simply trivial or bizarre objects (Sternberg and Lubart, 1999).

^6^Unique mental abilities have been crucial for evolutionary success of Homo sapiens and for the development of his complex social organization. However, these abilities have also become a target for mental disorders which often result in a reduced fitness and in conflicts between the individual and the conventions of society. To account for this evolutionary maladaptation, we have advanced a new concept: that of “mis-exaptation,” derived from S. J. Gould and E Vrba’s concept of exaptation. Mis-exaptation is a characteristic which, although it may confer positive effects in one field of activity, may reach an inappropriate degree of specialization to have deleterious effects in that or in another field thereby leading to a decrease in fitness of the individual (Agnati et al., 2012b).
